# The Interplay between Ca^2+^ Signaling Pathways and Neurodegeneration

**DOI:** 10.3390/ijms20236004

**Published:** 2019-11-28

**Authors:** Rodrigo Portes Ureshino, Adolfo Garcia Erustes, Taysa Bervian Bassani, Patrícia Wachilewski, Gabriel Cicolin Guarache, Ana Carolina Nascimento, Angelica Jardim Costa, Soraya Soubhi Smaili, Gustavo José da Silva Pereira

**Affiliations:** 1 Department of Biological Sciences, Universidade Federal de São Paulo, SP 09961-400 Diadema, Brazil; taysa_bassani@yahoo.com.br (T.B.B.); wachilewski@yahoo.com.br (P.W.); 2 Department of Pharmacology, Universidade Federal de São Paulo, SP 04044-020 São Paulo, Brazil; adolfo.erustes@gmail.com (A.G.E.); gabriel.cicolin@outlook.com (G.C.G.); anacarolina.acn@gmail.com (A.C.N.); angelicajardimcosta@gmail.com (A.J.C.); soraya.smaili23@gmail.com (S.S.S.); jspereira.gustavo@gmail.com (G.J.d.S.P.)

**Keywords:** Ca^2+^ signaling, intracellular organelles, plasma membrane, inositol-1,4,5-receptors, ryanodine receptors, ionotropic receptors, metabotropic receptors, TRP channels, two-pore channels, STIM and Orai, neurodegenerative diseases, autophagy, apoptosis

## Abstract

Calcium (Ca^2+^) homeostasis is essential for cell maintenance since this ion participates in many physiological processes. For example, the spatial and temporal organization of Ca^2+^ signaling in the central nervous system is fundamental for neurotransmission, where local changes in cytosolic Ca^2+^ concentration are needed to transmit information from neuron to neuron, between neurons and glia, and even regulating local blood flow according to the required activity. However, under pathological conditions, Ca^2+^ homeostasis is altered, with increased cytoplasmic Ca^2+^ concentrations leading to the activation of proteases, lipases, and nucleases. This review aimed to highlight the role of Ca^2+^ signaling in neurodegenerative disease-related apoptosis, where the regulation of intracellular Ca^2+^ homeostasis depends on coordinated interactions between the endoplasmic reticulum, mitochondria, and lysosomes, as well as specific transport mechanisms. In neurodegenerative diseases, alterations-increased oxidative stress, energy metabolism alterations, and protein aggregation have been identified. The aggregation of α-synuclein, β-amyloid peptide (Aβ), and huntingtin all adversely affect Ca^2+^ homeostasis. Due to the mounting evidence for the relevance of Ca^2+^ signaling in neuroprotection, we would focus on the expression and function of Ca^2+^ signaling-related proteins, in terms of the effects on autophagy regulation and the onset and progression of neurodegenerative diseases.

## 1. Introduction

As a key second messenger, Ca^2+^ activates several signaling molecules and participates in a variety of physiological and pathological processes. Signaling pathways utilizing Ca^2+^ are present during all stages of life, from egg fertilization to death, and have also been implicated in several pathologies and aging. Indeed, Ca^2+^ plays a central role in muscle contraction, cell proliferation, differentiation, metabolism, and death, as well as autophagy and other processes. However, the connection between Ca^2+^ signaling and central nervous system (CNS) protection remains elusive.

In neurons, Ca^2+^ mediates essential signaling processes and plays an important role in synaptic plasticity. In fact, the regulation of Ca^2+^ concentrations in each compartment is a prerequisite for normal neuronal function. The neuronal Ca^2+^ signaling machinery is able to simultaneously activate different Ca^2+^-dependent processes, even from a distance. Due to the fact that neurons are susceptible to slight perturbations in intracellular Ca^2+^ levels, regulating these levels can be viewed as neuroprotective.

There are several circumstances where altered Ca^2+^ concentrations, in an acute or chronic manner, lead to catastrophic cellular consequences, thus demonstrating the role this key second messenger has in controlling cell fate. In the present review, we discussed the role of Ca^2+^ signaling in neurodegenerative diseases, highlighting the interplay between the proteins altered in those pathological conditions and the subsequent alterations in Ca^2+^ regulation. A better understanding of these processes is directly relevant to the onset and progression of neuronal cell death.

### 1.1. A Brief Overview of Apoptosis and the Central Role of Mitochondria

Altered Ca^2+^ homeostasis and subsequent changes in the Ca^2+^ levels of cellular compartments can promote survival or induce the activation of cell death pathways [1,2]. Increased apoptosis is commonly observed with neuronal cell death associated with neurodegenerative diseases. It is a well-characterized type of programmed cell death that modifies cellular morphology, forms apoptotic bodies, and eventually results in complete phagocytosis. In neurodegeneration, there are two main apoptotic pathways, extrinsic and intrinsic. 

The extrinsic apoptotic pathway is induced by the activation of death receptors in the cellular membrane. For example, tumor necrosis factor receptor 1 (TNFR1) promotes the activation of initiator caspases (i.e., caspase 8) and activates effector caspases (i.e., caspase 3) (reviewed by [3]). On the other hand, the intrinsic pathway, also known as the mitochondrial pathway, has been shown to be activated by DNA damage, oxidative stress, and growth factor deprivation [4]. Additionally, some of the most important regulators of apoptosis are the members of the B-cell lymphoma protein 2 (Bcl-2) family, including pro-apoptotic (Bax, Bak, Bid) and anti-apoptotic (Bcl-2, Bcl-xL) proteins (reviewed by [5]).

During activation of the intrinsic pathway, Bax translocates from the cytosol to the outer mitochondrial membrane (OMM) [6], where it forms oligomers and leads to the formation of pores in the mitochondrial membrane, and decreases the mitochondrial membrane potential (ΔΨm), resulting in the release of pro-apoptotic molecules, such as cytochrome *c*, apoptosis-inducing factor (AIF), as well as other molecules stored in the intermembrane space (IMS) [7]. In the cytosol, cytochrome *c* binds to apoptotic protease activating factor 1 (APAF1), ATP/dADP, and procaspase 9, forming an apoptosome that subsequently activates effector caspases, with caspase 3 being especially susceptible to activation [3,8]. The extrinsic and intrinsic pathways can converge at caspase 8-mediated Bid cleavage, at which time the truncated Bid (tBid) is active and can translocate to the OMM, while Bax augments mitochondrial membrane permeabilization and apoptotic molecule release [9,10].

Alternatively, OMM permeabilization can also result from sustained mitochondrial permeability transition pore (mPTP) opening. Described by Hunter et al. (1976), the mPTP is a voltage-operated channel, located in the inner mitochondrial membrane (IMM) [11]. These pores are nonspecific to ionic and nonionic substrates and are opened in a transitory or sustained manner [12]. Under pathological conditions, sustained mPTP opening, also known as the high conductance state, increases reactive oxygen species (ROS) generation, promoting a massive release of Ca^2+^, nicotinamide adenine dinucleotide (NAD^+^), proteins, glutathione, and other metabolites into the cytosol (reviewed by [13]). In addition, the sustained opening can also promote morphological alterations to the mitochondria, resulting in reduced respiratory function, collapsed ΔΨm, and attenuated ATP synthesis (reviewed by [14]). These events lead to the release of pro-apoptotic factors, from the IMM, and intrinsic apoptosis pathway activation [15,16]. As would be discussed, mPTP opening is primarily regulated by increased Ca^2+^ concentrations in the mitochondrial matrix, oxidative stress, and reduced ΔΨm (reviewed by [17]), which can all contribute to neurodegenerative disease-mediated cell death. 

### 1.2. Is Ca^2+^ Unbalance Participating in Neurodegeneration?

Alzheimer’s disease (AD), Parkinson’s disease (PD), and Huntington’s disease (HD) are among the most prevalent neurodegenerative diseases. In the elderly population, AD is perhaps the most frequently diagnosed neurodegenerative disorder, progressively impairing the memory and learning processes. Most cases of AD and PD are sporadic and characterized by late-onset, mostly affecting people with 60 years of age or more; however, about 10% corresponds to familial cases, having an early onset and commonly observed in individuals that are around 50 years of age or younger. On the other hand, HD is an inherited monogenic autosomal dominant disease, with symptoms often appearing at 40–50 years of age.

Components associated with familial cases of neurodegenerative diseases that have been found to interfere with Ca^2+^ signaling include:

(1) AD: mutations in genes codifying amyloid precursor protein (APP) or Presenilins 1 or 2. Presenilins are part of the catalytic subunit of the γ-secretase complex. The γ- and β-secretase enzymes together cleave APP, consequently generating β-amyloid peptides (Aβ), subsequently forming protein aggregates.

(2) PD: the presence of intraneuronal protein aggregates called Lewy bodies, mainly composed of α-synuclein. Mutations in leucine-rich repeat kinase 2 (LRRK2) may stimulate protein activity.

(3) HD: mutations, present as an expansion of CAG trinucleotides (polyglutamine repeats) close to the N-terminus, of the protein huntingtin (mHtt), which are prone to aggregation.

Another inherited neurodegenerative disease involving protein aggregation includes frontotemporal dementia (FTD), which is caused by mutations in either the microtubule-associated protein Tau (MAPT:FTDP - 17MAPT) or the progranulin (PGRN:FTDP - 17PGRN) genes. Additionally, Creutzfeldt–Jakob disease (CJD) is associated with the accumulation and aggregation of a misfolded/unfolded isoform of brain cellular prion protein (PrP^c^), known as PrP^Sc^, resulting in neuroinflammation and neurodegeneration. A more thorough discussion related to these protein aggregation events and Ca^2+^ signaling would be discussed later in this review.

Moreover, it is well known that disruptions in Ca^2+^ homeostasis can alter neuronal activity. Several studies reported that Ca^2+^ signaling dysfunction is involved in aging and neurodegenerative processes, promoting abnormalities in synapses, mitochondrial function, and inducing endoplasmic reticulum (ER) stress. For example, PrP^Sc^ accumulation activates an adaptive response, known as the unfolded protein response (UPR), to correct improperly folded ER proteins [18]. Thus, it should come as no surprise that intracellular Ca^2+^ concentrations are tightly regulated and are essential for neuronal function and survival, with Ca^2+^ overload being related to excitotoxic neuronal cell death. In the following sections, the mechanisms by which Ca^2+^ promotes cell death and the effect of neurodegenerative disease-related proteins on ionic buffering would be discussed.

## 2. The Role of Ca^2+^ Signaling Components in Neurodegenerative Diseases

### 2.1. Plasma Membrane Ca^2+^ Channels

The regulation of Ca^2+^ levels is responsible for the selective activation of Ca^2+^-mediated signal transduction pathways. Neurons can modulate the activity of pumps, as well as voltage and/or ligand-gated ion channels, to generate Ca^2+^ fluxes through the plasma membrane. The activation of voltage-gated Ca^2+^ channels (VGCC) promotes Ca^2+^ influx with the electrochemical gradient, resulting in increased cytoplasmic Ca^2+^ concentration-dependent enzyme activation, neurotransmitter release, neurite outgrowth, hormone secretion, and other Ca^2+^-dependent processes.

The VGCCs participate in several cellular processes, including hormone secretion, muscle contraction, and gene expression. They are classified into two major channel categories: high voltage-activated (L-, P-, and Q-type channels) and low voltage-activated (T-type channels), with the α1 subunit determining the Ca^2+^ channel subtype. The L-type Ca^2+^ channels participate in Ca^2+^-induced long-term potentiation in dendritic spines and postsynaptic dendrites, while P- and Q-type Ca^2+^ channels, present in the presynapse, generate inward Ca^2+^ currents and initiate neurotransmitter release and presynaptic plasticity. The opening of T-type Ca^2+^ channels results in currents that induce the rhythmic action potential production in the sinoatrial node of the heart (reviewed by [19]).

There is reported evidence that VGCC activity is altered in AD. For example, Aβ has been shown to stimulate Ca^2+^ influx through L-type VGCCs in a variety of cellular models (reviewed by [20]), an effect that could be blocked by nimodipine [21], and Aβ_1-42_ oligomers have been reported to suppress P/Q-type VGCCs Ca^2+^ influx, consequently impairing spontaneous synaptic activity [22]. Interestingly, Aβ oligomers have been shown to activate VGCC more strongly than aggregated forms of the peptides, suggesting that aggregation state could differentially regulate VGCC activity [23]. In FTD, which is a Tau-related disease, a case report has associated elevated levels of P-, Q-, and N-type Ca^2+^ channels antibodies with a short onset of FTD symptoms in a middle-aged patient, suggesting the involvement of VGCC in this pathology [24].

Substantia Nigra *pars compacta* (SNpc) neurons produce action potentials without the need of synaptic input, setting them apart from most other types of neurons [25]. A previous study showed that, in PD models, constitutive L-type Ca_V_1.3 channel activity made SNpc cells more susceptible to Ca^2+^-mediated excitotoxicity, caused by subjacent mitochondrial dysfunction [26]. Furthermore, the electrical activity of SNpc neurons requires an oscillating Ca^2+^ influx and low levels of intracellular Ca^2+^-buffering proteins, such as calbindin; thus, slight increases in Ca^2+^ levels could have potentially damaging results [27].

In the α-synuclein A53T transgenic mouse model for PD, the aggregate-prone mutant protein has been associated with the upregulation and hyperactivation of N-type Ca^2+^ channels, which contributes to the increased susceptibility of SNpc neurons to cell death [28]. During aging, Cav1.3 L-type Ca^2+^ channel activity is increased in SNpc neurons. Remarkably, in an in vivo model of PD, the pharmacological inhibition of Cav1.3 L-type Ca^2+^ channels, with isradipine, restores the normal activity of dopaminergic cells, protecting SNpc neurons from cell death [29].

The VGCCs have also been shown to be sensitive to dihydropyridines, which are commonly used to treat hypertension. Additionally, amlodipine, an L-type VGCC blocker, is being tested in a clinical trial (NCT02913664), as a drug candidate that can reduce the risks for developing AD, and nilvadipine, an L-type VGCC antagonist, has recently completed a phase III trial as a treatment for AD (NCT02017340). In PD, some dihydropyridine compounds have been shown to reduce the overall population risk for developing PD [30,31], and clinical trials evaluating the efficacy of isradipine in early-onset PD are currently underway (NCT02168842).

**Figure 1:**
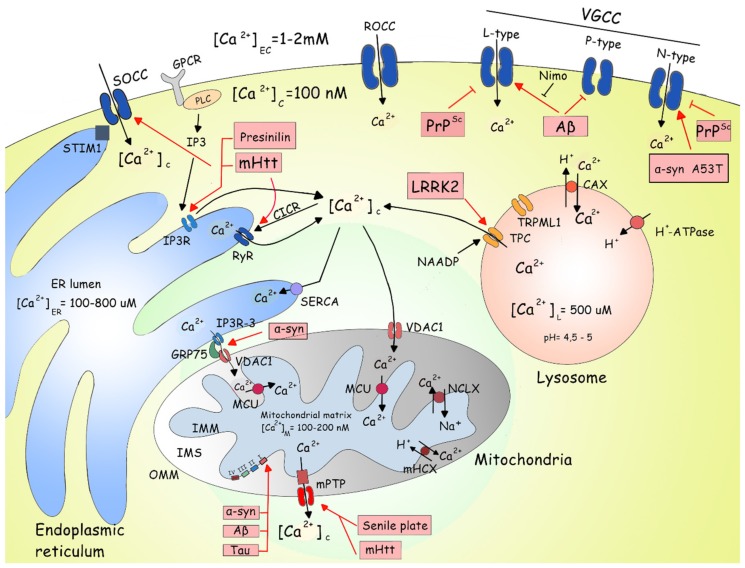
Crosstalk between proteins associated with neurodegenerative diseases and Ca^2+^ physiology. The cellular Ca^2+^ homeostasis is regulated by a synchronized system of pumps and channels, located in plasma membranes of cells and organelles. The influx of calcium (Ca^2+^) from the extracellular space is mediated by voltage-gated Ca^2+^ channels (VGCC), including L-type, P-type, and N-type channels. Dysfunctions of these channels have been described in Parkinson’s disease (PD), where the A53T mutation in α-synuclein upregulates and hyperactivates N-type channels. In Alzheimer’s disease (AD), β-amyloid peptide (Aβ) stimulates L-type channels, which can be blocked by nimodipine. In prion disease, prion protein (PrP^Sc^) can inhibit the L-type channel while reducing the activity of the N-type channel. The store-operated Ca^2+^ channel (SOCC) mediates Ca^2+^ influx in response to reduced intracellular stocks. Mutant huntingtin (mHtt) promotes SOCC hyperactivation, resulting in increased Ca^2+^ entry through the channel. The Ca^2+^ level in the endoplasmic reticulum (ER) is controlled by stromal interaction molecule 1 (STIM1), which under low ER Ca^2+^ concentrations forms dimers and interacts with the ORAI channel, promoting the influx of Ca^2+^ to the cytosol. Many receptors are bound to G protein complexes (GPCR) activating phospholipase C (PLC) and cyclic adenosine monophosphate (AMPc), promoting the synthesis of inositol-1,4,5-triphosphate (IP_3_), which upon binding to the inositol-1,4,5-triphosphate receptor (IP_3_R) in the ER membrane, promotes the release of ER Ca^2+^ to the cytosol. Increased cytosolic Ca^2+^ levels also activate the ryanodine receptor (RyR), releasing Ca^2+^ by a phenomenon called Ca^2+^-induced Ca^2+^ release (CICR). In Huntington’s disease, mHtt directly influences IP_3_R and RyR activities, increasing cytosolic Ca^2+^ concentrations and decreasing the levels of this ion in the ER. Mutant presenilin, associated with AD, can also modulate the activity of IP_3_R, increasing the Ca^2+^ release by this channel. Sarcoendoplasmic reticular Ca^2+^ ATPase (SERCA) mediates the transport of Ca^2+^ back to the ER lumen. Contact sites between the ER and mitochondria are called mitochondrial-associated ER membranes (MAM), which also represent a region of intense Ca^2+^ traffic. IP_3_R and voltage-dependent anion channel 1 (VDAC1) are the main channels involved in MAM Ca^2+^ transport. Initially, Ca^2+^ stored in the ER is released through IP_3_R, diffuses through the MAM, is taken up by the VDAC, located in the outer mitochondrial membrane (OMM), and transported to the mitochondrial matrix by the mitochondrial Ca^2+^ uniporter (MCU). The mitochondrial chaperone glucose-related protein 75 (GRP75) physically interacts with both channels and facilitates Ca^2+^ transport. In PD, it has been proposed that α-synuclein could be inserted in the MAM, but its pathological role has yet to be elucidated. In the mitochondria, the influx of Ca^2+^ is mediated by VDAC and MUC, as mentioned above, and the efflux is mediated by Ca^2+^ exchangers, Na^+^/Ca^2+^ exchanger (NCLX), and mitochondrial H^+^/Ca^2+^ exchanger (mHCX), in the inner mitochondrial membrane (IMM). The controlled opening and closing of the mitochondrial transition pore (mPTP) also mediates the Ca^2+^ efflux from the mitochondria. However, sustained mPTP opening has been linked to apoptosis. The senile plates and mHtt can affect the mPTP, consequently altering the activity of this pore. In mitochondria, the α-synuclein, Aβ, and Tau can directly impair the complex I activity. Lysosomes are another important organelle directly involved in Ca^2+^ signaling and homeostasis and express a variety of Ca^2+^ channels, including transient receptor potential (TRP) and two-pore channels (TPCs). Nicotinic acid adenine dinucleotide phosphate (NAADP) activates TPC, promoting the release of Ca^2+^ from lysosomes, which is amplified by CICR via RyR activation. In PD, mutations in leucine-rich repeat kinase 2 (LRRK2) leads to the upregulation of the protein and increased Ca^2+^ release through TPC. The Ca^2+^/H^+^ exchanger (CAX) mediates the influx of Ca^2+^ in lysosomes, and H^+^-ATPase promotes lysosomal acidification. The red arrows demonstrate the pathological stimulus and influence of each protein in Ca^2+^ channels and pumps. The black arrows show the physiological paths of cellular Ca^2+^ homeostasis.

With regard to prion diseases, PrP^c^ has been associated with VGCC dysfunctions. A synthetic PrP fragment (PrP106–126) has been found to inhibit L-type Ca^2+^ channel activity in rat cerebellar granule cells, decreasing Ca^2+^ currents, and leading to apoptosis [32]. Also, gonadotropin-releasing hormone neuronal cells (ScGT1-1) infected with PrP^Sc^ has presented N-type Ca^2+^ channel dysfunction, thus attenuating Ca^2+^ responses [33]. Taken together, these data provide evidence that neurodegenerative disorder-related intracellular proteins are capable of regulating Ca^2+^ influx via VGCC, which could potentially impair Ca^2+^ homeostasis (Figure 1).

### 2.2. Plasma Membrane Receptors

Physiologically, many stimuli can cause intracellular Ca^2+^ concentrations to rise. For example, the activation of metabotropic receptors—in the plasma membrane—results in Ca^2+^ release from the ER via inositol-1,4,5-triphosphate (IP_3_) receptors [34]. Glutamate is one of the most versatile neurotransmitters in the CNS and is capable of activating both ionotropic and metabotropic receptors, with three groups of metabotropic glutamatergic receptors (mGluR) having been identified. Group I (mGluR1 and mGluR5) is coupled to Gq protein and results in increased IP_3_ levels, group II (mGluR2 and mGluR3) and group III (mGluR4 and mGluR6–8) are coupled to Gi protein and results in decreased cyclic AMP (cAMP) levels. Pathologically, Aβ_42_ oligomers, but not monomers, alter Ca^2+^ release from ER stores, via mGluR5. It was also previously reported that Aβ_42_ oligomers reduced Ca^2+^ release induced by IP_3_ and increased caffeine-induced ryanodine receptor (RyR) Ca^2+^ release without altering the total Ca^2+^ content in the intracellular stores [35].

Glutamate primarily acts on ionotropic membrane receptors, such as N-methyl-D-aspartic acid (NMDA), β-amino-3-hydroxy-5-methylsoxazole (AMPA), and kainate receptors. The NMDA receptors (NMDAR) are composed of NR1 and NR2A-D subunits, and during action potential generation, there is a rapid depolarization resulting in a high, transient Ca^2+^ peak, primarily due to contributions from the AMPA and kainate receptors, which exhibit fast kinetics and rapid (millisecond) desensitization [36]. It is well known that NMDAR is stimulated by glutamate, with glycine acting as a co-agonist [37]. However, as the membrane potential increases, there is a decrease in the affinity for this ion, and inhibition is attenuated. In vitro experiments with cortical neurons demonstrated that Aβ oligomers could increase intracellular Ca^2+^ levels by activating, through the N2B subunit, NMDAR [38]. Additionally, in cultured cortical neurons, Aβ promotes NMDAR endocytosis and reduces NMDAR expression in vivo, using an AD mouse model. Together, these results represent potential Aβ-mediated mechanisms for the inhibition of glutamatergic transmission and synaptic plasticity [39]. It has also been reported that in AD and during normal aging, NMDAR hypofunction promotes Ca^2+^ dysregulation and increases oxidative stress, consequently impairing synaptic plasticity [40]. 

Glutamate receptors are essential for neuronal plasticity, participating in long-term potentiation (LTP) and long-term depression (LTD) [41]. This synchronism between adjacent cells (neurons or glial cells) and the target neuron results in the promotion of sustained postsynaptic depolarization, terminating with a molecular cascade of protein production and neuritogenesis [42], which is essential for memory consolidation. In order to regulate Ca^2+^ signaling in the spine, the coordinated enzymatic activities of Ca^2+^calmodulin-dependent kinase II (CaMKII) and calcineurin (CaN)—a Ca^2+^-dependent phosphatase—are involved. In synapses, CaMKII and CaN are expressed at high levels, with slight alterations in Ca^2+^ concentrations, resulting in reduced CaMKII autophosphorylation and increased CaN activity, thus shifting the balance of these enzyme activities. Synaptic CaMKII is essential for LTP, being centrally involved in memory formation, whereas CaN activation is necessary for LTD processes in the hippocampus. In AD, alterations in Ca^2+^ signaling have been suggested to shift the synaptic activities of CaMKII and CaN, favoring CaN and disrupting the equilibrium between LTP and LTD mechanisms. Due to the subsequent LTD stimulation and LTP inhibition, synapses are lost in AD brains, resulting in memory impairment (reviewed by [43]).

The hyperactivation of glutamate receptors, mainly NMDAR, can inflict cell injury and, in some cases, result in cell death [44], a process known as excitotoxicity. Previous work has shown that massive and prolonged Ca^2+^ influx leads to apoptotic cell death, induced by increased ROS generation, sustained mPTP opening, and the release of pro-apoptotic signals [45]. It is plausible that NMDAR could be overactivated during the early stages of AD since this receptor is a potential source for intracellular Ca^2+^ and an essential component of excitatory synapses [46]. More recently, glutamatergic nervous system degeneration related to excitotoxicity was reported in *Caenorhabditis elegans* expressing a mutant form of Tau, 2N4R-Tau^A152T^ (Tau^AT^). In this model, Tau^AT^ expression increased cytosolic Ca^2+^ concentrations, due to release from ER stores, hyperactivated CaN, and initiated necrosis [47], thus providing evidence for the participation of Tau variants in the modulation of Ca^2+^ signaling. Furthermore, in FTD, the involvement of AMPA receptors (AMPAR) has been associated with disease-related presenile onset due to the presence of anti-GluA3 (AMPAR) antibodies in the sera of patients, which promote dendritic spine loss in culture [48]. Another study performed a broad genetic interaction map, using data acquired from Italian FTD patients, and revealed that there was an association with GRIN2B (a component of the NR2 subunit of NMDAR), meaning that alterations in Ca^2+^ signaling could contribute to neuronal damage [49].

With regards to PD, in an experimental dopaminergic denervation model, increased cortico-striatal glutamatergic activity was observed [50]. In addition, a reduction in NMDA and AMPA binding sites was found to occur in PD models produced with 6-OHDA dopaminergic lesions [51], perhaps as a compensatory mechanism for the excitotoxic stimuli. Additionally, dopamine inhibits acetylcholine release [52] and modulates glutamate release in the striatum (a PD-related structure), and in its absence, glutamatergic synapse hyperactivation in the motor cortex occurs [53]. It has been demonstrated that in the striatum of senescent animals, Ca^2+^ homeostasis is altered when challenged with this excitatory amino acid [54].

There is evidence that indicates there is a striatal synaptic dysfunction in HD that is related to alterations in Ca^2+^ signaling [55]. For example, the expression of mHtt has been shown to increase the vulnerability of striatal medium spiny neurons (MSNs) to glutamate-induced excitotoxicity in a transgenic HD mouse model [56]. It is likely that mHtt induces enhanced surface expression of functional NR1/NR2B NMDAR subtypes [57] since increased NMDAR Ca^2+^ currents have been associated with fast receptor translocation to the cell membrane [58]. As a result of the altered NMDA currents, an imbalance between pro-survival/synaptic and pro-death/extrasynaptic NMDAR activity might occur in the MSNs and could contribute to HD pathogenesis (reviewed by [59]). In extrasynaptic adult neurons, NMDARs containing N2B subunits mediate the potentiation of NMDAR currents [60]. Consistent with these findings, memantine, an NMDAR inhibitor, provided neuroprotection in an in vitro HD model [61], as well as in a transgenic HD mouse model, in which low doses of the drug attenuated protein inclusions and improved behavior [62,63]. However, at higher doses, memantine exacerbates these outcomes, possibly due to extrasynaptic and synaptic NMDAR inhibition [63], suggesting that excessive extrasynaptic NMDAR activity in the striatum could accelerate HD-related synaptic loss. It should also be pointed out that this NMDAR inhibitor showed promising effects in a small-scale clinical trial that enrolled HD patients [64].

Besides glutamatergic signaling, cholinergic neurons are also associated with the cognitive impairments observed in AD patients [65], where Aβ oligomers have been shown to inhibit cholinergic transmission [66,67]. The interactions between Aβ and subunits of the nicotinic acetylcholine receptors (nAChR) have been demonstrated, as reported in brain samples of AD patients, where only the α7 subunit of nAChR co-localizes and co-immunoprecipitates with the Aβ_1-42_ [68,69]. Additionally, α7 also immunoprecipitates with Aβ_1-42/40_ in a transgenic mouse model [70], and that oligomeric Aβ_1-40_ promotes α7 nAChR activation in a neuroblastoma cell line [71]. Another study showed that stimulating the receptor with Aβ and nicotine led to activation, resulting in perturbed calcium homeostasis, mitochondrial dysfunction, and oxidative stress [72]. Finally, in samples from the hippocampus and cortex of rats, the addition of Aβ_1-42/42_ induced the activation of both α7 and non-α7, and increased Ca^2+^ influx [73].

In prion disease, Fang et al. (2018) demonstrated that PrP^Sc^ initiated a synaptotoxic signaling cascade that activates NMDA and AMPA receptors in hippocampal neurons, increases intracellular Ca^2+^ concentrations, stimulates p38 mitogen-activated protein kinase (MAPK), and depolymerizes actin filaments in dendritic spines, leading to the collapse of the cytoskeleton [74]. Synaptic degeneration is restricted to excitatory postsynaptic neurons, resulting in impaired synaptic transmission. Consistent with these findings, De Mario et al. (2017) showed that PrP^c^ protected neurons from Ca^2+^ overload via the modulation of ionotropic glutamatergic receptors function [75]. In addition, in the peripheral nervous system, PrP^c^ was reported to promote axonal growth in a Ca^2+^-dependent manner [76]. PrP^c^ was shown to form a complex with mGluR5, and this complex could be activated by ligands, such as β-amyloid oligomers and laminin, promoting Ca^2+^ influx into the neurons [77]. In fact, a mutant PrP^c^ induced abnormal Ca^2+^ currents in cultured neurons and cerebellar slices, resulting in glutamate-induced excitotoxicity and neuronal death [78].

### 2.3. Intracellular Ca^2+^ Stores in Physiology and Neurodegenerative Diseases

#### 2.3.1. Endoplasmic Reticulum (ER)

The ER is the largest organelle in a cell and is responsible for a variety of functions, ranging from Ca^2+^ storage, lipid and protein biosynthesis, secretion, and transport [79]. Numerous factors can affect normal ER function, including enhanced protein synthesis, increased unfolded and/or misfolded protein content, perturbed redox regulation in the lumen, and altered Ca^2+^ concentrations in response to increased levels of the ion in the cytosol [80]. In addition to synthesizing proteins, the ER is also responsible for making modifications and folding nascent polypeptides, playing a critical role in protein quality control mechanisms, including the unfolded protein response (UPR) [81]. Notably, many neurodegenerative diseases are characterized by the accumulation of misfolded proteins, with protein aggregates affecting normal cell function in AD, PD, and HD (Figure 1).

With regards to Ca^2+^ homeostasis in the ER, extracellular Ca^2+^ concentrations range from 1–2 mM, cytosolic is about 100 nM, and the ER lumen is 100–800 μM [82,83]. It is known that the stimulation of the IP_3_ receptor (IP_3_R), in the ER membrane, promotes Ca^2+^ release from the ER to cytosol, but other Ca^2+^ homeostasis mechanisms also exist in the ER (reviewed by [84]). For example, Ca^2+^-mediated ryanodine receptor (RyR) activation leads to Ca^2+^-induced Ca^2+^ release (CICR) [85], and activation of the sarcoendoplasmic reticular Ca^2+^ ATPase (SERCA), a pump that mediates the transport of Ca^2+^ back to the lumen of ER, represents a Ca^2+^ reuptake mechanism (reviewed by [84]). Furthermore, the Ca^2+^ sensor stromal interaction molecule 1 (STIM1) and plasma membrane protein ORAI1 assemble to form the selective pore of store-operated Ca^2+^ channel (SOCC) and increase cytoplasmic Ca^2+^ concentrations when the ER stores are depleted (reviewed by [59]). 

Previously, we discussed the impact of Aβ and Tau alterations on Ca^2+^ signaling in AD. Additionally, mutant presenilins have also been implicated in AD-related Ca^2+^ dysregulation, exerting similar effects through different mechanisms. In the ER, presenilins have been proposed to function as Ca^2+^-leak pores, and several familial AD mutations may compromise the pore function, leading to Ca^2+^ accumulation in the ER [86,87]. These mutant presenilins likely modulate IP_3_R activity, consequently augmenting channel-mediated Ca^2+^ release [88,89]. Presenilin 1 was also reported to increase the expression levels and single-channel activity of RyR [90]. Previous work with presenilin 1 and 2 knockout mice showed that these animals presented reduced SERCA activity and increased cytosolic Ca^2+^ due to physical interactions between these proteins and SERCA [91]. Recently, presenilins 1 and 2 mutations resulted in reduced Ca^2+^ influx via SOCC and low levels of STIM1, indicating that mutant presenilins could dysregulate Ca^2+^ homeostasis [92].

There is evidence that supports the role of dysregulated RyR function in AD, caused by excessive Ca^2+^ release from the ER into the cytoplasm [93]. For example, when compared to controls, post-mortem hippocampal samples from individuals with early-stage AD presented increased [H]^3^ryanodine binding, which is indicative of upregulated RyR protein expression [94]. In the young, adult, and aged neurons from presenilin mutant mice, RyR activity is enhanced, and Ca^2+^ release is increased [95]. In prion disease models, cortical neurons treated with synthetic PrP and Aβ peptides exhibited increased cytosolic Ca^2+^ contributions, mediated through RyR and IP_3_R, ultimately leading to ER stress and apoptosis [96].

Neurotoxins, such as 1-methyl, 4-phenyl pyridinium (MPP+) and salsolinol, used for pharmacological PD models, were found to downregulate transient receptor potential canonical l (TRPC1) expression and induce thapsigargin-mediated Ca^2+^ influx, consequently resulting in SH-SY5Y cell degeneration in vitro. Conversely, the overexpression or activation of TRPC1 protected neurons against the effects of the neurotoxin [97,98]. Studies involving PARK14, a gene related to familial PD and codifies for the Ca^2+^-independent phospholipase A2 group 6 (PLA2g6) protein, have also been associated with altered Ca^2+^ homeostasis. Interestingly, skin fibroblasts obtained from idiopathic and familial PD patients with the PLA2g6^R747W^ mutation displayed reduced Ca^2+^ entry via SOCC and depleted Ca^2+^ stores in the ER. Inhibition of PLA2g6-dependent Ca^2+^ signaling in a mouse model, using genetic tools, triggered autophagic dysfunction in and death of dopaminergic SNpc neurons [99].

The ER Ca^2+^ stores are also indirectly influenced by mHtt. For example, mHtt has been shown to bind to IP_3_R1, increasing the affinity of the receptor for IP_3_, and leaking Ca^2+^ ions from the ER [100]. Additionally, mHtt may also bind to RyR, causing excessive Ca^2+^ leakage through the receptor and depleting internal ER Ca^2+^ stores [101]. In HD, spiny neurons expressing mHtt exhibit enhanced neuronal SOCC-mediated Ca^2+^ influx due to sensitization of IP_3_R to IP_3_ agonists in the ER, consequently leading to continuous ER Ca^2+^ store depletion. In an attempt to regulate Ca^2+^ influx via SOCC, there is an upregulation of STIM2 [102] and TRPC1 [103] expression in the ER and plasma membrane, respectively. Collectively, these findings indicate HD-associated neurodegeneration is, at least partially, mediated by responses to ER Ca^2+^ depletion.

The mitochondria and ER play important roles in cellular Ca^2+^ homeostasis, with intense ion exchange occurring through mitochondria-associated ER membranes (MAM). This ER-specific domain is enriched with cholesterol and anionic phospholipids, allowing the ER and OMM to become very close (10–30 nm) (reviewed by [104]), and functioning as a region for continuous communication and ion transfer between these organelles. In addition to the unique lipid combination, these regions are also enriched with relevant Ca^2+^ channels and regulators. For example, the IP_3_R is the main channel that mediates Ca^2+^ efflux from the ER to the cytosol, and the voltage-dependent anion channel (VDAC) is the primary channel for transporting Ca^2+^ from the OMM to the IMM, and subsequently taken up into the mitochondrial matrix by mitochondrial Ca^2+^ uniporter (MCU) [105]. Following IP_3_R activation, Ca^2+^ stored in the ER is released and travels across the MAM, and taken up by VDAC in the OMM of the mitochondria [106]. In terms of pathology, it has been suggested that normal levels of α-synuclein have no effect on Ca^2+^ homeostasis and cell function. Furthermore, the protein deglycase DJ-1 and parkin play a role in the maintenance of ER-mitochondria contact sites, and parkin may also directly stimulate phospholipase Cγ1 (PLCγ1) in the cell membrane, ultimately leading to increased cytosolic Ca^2+^ concentrations (reviewed by [107]).

#### 2.3.2. Mitochondria

In addition to synthesizing large amounts of ATP, mitochondria also play essential roles in a variety of physiological and homeostatic processes, including bioenergetics, cell cycle regulation, apoptosis, and Ca^2+^ regulation (reviewed by [108]). Many of these processes occur because mitochondria are multifunctional organelles, with a double-membrane structure that generates and maintains an electrochemical gradient of protons (H^+^), through the coordinated activities of enzyme complexes of the electron transport chain (ETC), located in the IMM (reviewed by [109]).

Interestingly, many functions of the mitochondria are directly linked to Ca^2+^ storage and flux in the organelle, which can influence the expression and activity of several enzymes and carriers. In the IMS, FAD-glycerol phosphate dehydrogenase (GPDH) has been shown to be regulated by changes in Ca^2+^ levels [110]. While in the mitochondrial matrix, increased Ca^2+^ concentrations stimulate enzymes that participate in the tricarboxylic acid cycle (TCA). In addition, components of the ETC are also modulated in response to mitochondrial Ca^2+^ levels. In fact, it has been shown that increased Ca^2+^ levels stimulate, by about two-fold, the activities of ETC complexes I, III, and IV [111]. Moreover, Ca^2+^ has also been shown to modulate the activity of F_0_F_1_-ATP synthase [112].

Mitochondria gained notoriety in HD pathogenesis when it was discovered that systemically administering a mitochondrial complex II inhibitor, 3-nitropropionic acid (3-NP), to rats caused preferential striatal degeneration and impaired energy metabolism, resembling characteristics of the neurodegenerative disease [113]. Additionally, disturbances in the activities of ETC complexes II, III, and IV were detected in the caudate nucleus of striatum from HD patients [114]. Due to the observed weight loss, even with adequate caloric intake, individuals with HD appear to suffer from a global metabolic dysfunction [115,116]. Consistent with this hypothesis, lymphoblasts isolated from HD patients presented reductions in ATP/ADP ratios, which were indicative of systemic metabolic changes and proportional to the mHtt glutamine repeat length [117]. 

The transport of Ca^2+^ across the OMM is mediated by VDAC1, a channel permeable to ions and small hydrophilic molecules, of which some are related to metabolic functions, such as succinate, malate, pyruvate, NADH, ATP, ADP, and phosphate [109,118]. As mentioned above, Ca^2+^ concentration changes in the IMS can impact the activity of some metabolic enzymes. Finally, to reach the mitochondrial matrix, Ca^2+^ must cross the IMM, which is impermeable to the ion. This transport step is achieved using the ΔΨm to create a driving force for Ca^2+^ accumulation in the matrix. However, any factor that can cause the ΔΨm to collapse will consequently reduce and/or suppress mitochondrial Ca^2+^ uptake [106]. Compared to the initial steps of Ca^2+^ entry into the mitochondria, the movement of Ca^2+^ through the IMM is more complex and requires the specialized MCU. This IMM protein modulates mitochondrial Ca^2+^ uptake when concentrations of this ion are elevated in the cytosol [119]. The MCU is a highly selective Ca^2+^ channel, which strongly binds to the ion and transports it against the electrochemical gradient and into the mitochondrial matrix [105]. In vitro experiments demonstrated that silencing MCU1 expression promoted the reduction of Ca^2+^ levels in the mitochondrial matrix and that the overexpression of this protein caused a notable accumulation of this ion [120].

It is possible that Aβ inhibits the transport of nuclear-encoded proteins into the mitochondria since interactions with the translocase of the outer mitochondrial membrane 40 (TOM40) complex reduce the import of complex IV subunits. Moreover, Aβ interferes with the function of ETC complexes IV and V, while Aβ and Tau act synergistically to impair complex I function. The accumulation of Aβ in the IMS has also been shown to compromise the permeability of both the IMM and OMM (reviewed by [121]).

Neurotoxins, such as MPP+, induce SH-SY5Y cell death by activating the intrinsic apoptosis pathway, which is highly dependent on mitochondria. Additionally, it appears that MCU plays a role in MPP+-induced cell death since MCU overexpression partially attenuates neuronal cell death, and downregulation results in augmented autophagy and cell death due to AMPK activation, thus demonstrating that disruptions in mitochondrial Ca^2+^ homeostasis can contribute to neuronal degeneration in an in vitro model of PD [99]. Furthermore, mitochondrial dysfunctions can also be mediated by α-synuclein, which binds to ETC complex I, consequently compromising the normal function of this enzyme [122].

Previous studies have shown that Aβ can trigger the opening of the mPTP by interacting with and translocating cyclophilin D (CypD), a key component of the mPTP, adenine nucleotide translocase (ANT), and VDAC to the IMM (reviewed by [121]). In AD, the formation of senile plaques can directly affect mPTP opening, promoting the depolarization of mitochondria [123]. In addition, studies have suggested that the mPTP could be involved in the observed metabolic stress associated with AD [17,124,125]. In an AD model, using APP overexpression, knocking out CypD enhanced cognitive behavior, attenuated neuronal loss, and prevented mitochondrial dysfunction [124,126]. Recently, it was reported that AD patient-derived fibroblasts exhibited signs of mitochondrial Ca^2+^ dysregulation when compared with normal fibroblasts. For example, fibroblasts from AD patients exhibited long-lasting mPTP activation, and pharmacological blockage with Cyclosporine A (CsA) improved mitochondrial and cytosolic Ca^2+^ dysregulation. Additionally, the AD fibroblasts displayed a pattern of mitochondrial dysfunction that resembled what is observed in AD brains, thus highlighting the potential utility in employing fibroblasts as peripheral biomarkers for early AD detection [127]. The opening of the mPTP has also been observed in PD animal models following rotenone treatment [128] and, in HD models, through mHtt expression [17].

Several findings suggest that mHtt interacts with the IMM and OMM through its cytosolic N-terminus. This interaction might have a negative impact on protein import [129] and could precede alterations in the mitochondrial Ca^2+^ [130]. Additionally, it has been reported that mHtt strongly associates with mitochondrial proteins [131], and studies using HD animal models suggest that mHtt directly stimulates mPTP opening and cytochrome *c* release, even at lower Ca^2+^ concentrations, and triggers neuronal apoptosis [132]. Although the causative role of mitochondrial dysfunction is not definitive, it can preferentially damage striatal neurons in a manner that is consistent with HD pathogenesis.

#### 2.3.3. Acidic Organelles

Besides the ER and mitochondria, Ca^2+^ is also stored in a variety of acidic organelles, including lysosomes, lysosome-related organelles, endosomes, secretory vesicles, vacuoles, and Golgi complex [133,134], with concentrations of approximately 0.5 mM [135]. Depending on cell type, the intercellular pH of these acidic Ca^2+^-stores ranges from pH 4–5, with the stored Ca^2+^ playing a central role in intracellular Ca^2+^ signaling processes, hydrolase activity, and autophagy regulation [136]. These organelles express several types of Ca^2+^-permeable ion channels, including members of the transient receptor potential channel (TRP) and two-pore channel (TPCs) families [136].

The TRP family is divided into two groups, which are further divided into seven subfamilies of channels. Group 1 encompasses the TRPC (canonical), TRPV (vanilloid), TRPM (melastatin), TRPN (Drosophila NOMPC—no mechanoreceptor potential C), and TRPA (ankyrin) subfamilies, while Group 2 contains TRPP (polycystin) and TRPML (mucolipin) (reviewed by [137]). These channels display diverse cation selectivities and rely on specific activation mechanisms, involving voltage and temperature, as well as ligand-gated channels and those that are constitutively active [138]. The permeability to Ca^2+^ can vary considerably among TRP channels. While most display low selectivity for Ca^2+^ (P_Ca_/P_Na_ 0.3–10), TRPV5 and TRPV6 exhibit high Ca^2+^ selectivity (P_Ca_/P_Na_ > 100), and TRPM4 and TRPM5 are Ca^2+^ impermeable [138].

With regards to TPCs, these channels are voltage-gated non-selective cation channels, with two isoforms, TPC1 and TPC2, identified in humans [139]. These channels are physiologically activated by the Ca^2+^-mobilizing second messenger nicotinic acid adenine dinucleotide phosphate (NAADP) [140,141]. Initially, NAADP induces a local Ca^2+^ release from acidic Ca^2+^ stores that subsequently triggers amplified Ca^2+^ release from the ER, via IP_3_R and RyRs [142]. Due to the fact that TPCs mediate their physiological effects by stimulating Ca^2+^ release from acidic organelles, dysfunctions in these channels have been related to PD [143]. For example, mutations in leucine-rich repeat kinase-2 (LRRK2), a protein that interacts with and potentially modulates the function of several proteins in the endo-lysosomal compartment, including TPCs, are the most common cause of familial PD. Therefore, it is plausible that mutated LRRK2 could alter intracellular Ca^2+^ handling of acidic stores [144], and also regulate TPC2 function and perhaps intracellular vesicle trafficking, thus, at least partially, accounting for the observed LRRK2-related pathophysiology [145]. Hockey et al. (2015) showed, using fibroblasts isolated from PD patients, that the lysosomes were enlarged and aggregated [143]. Interestingly, the observed changes in lysosomal morphology were restored by genetic modification or pharmacological inhibition of TPC2, as well as by buffering local Ca^2+^ signals with BAPTA-AM (1,2-bis-(o-Aminophenoxy)-ethane-N,N,N′,N′-tetraacetic acid, tetraacetoxymethyl ester), which is a Ca^2+^ chelator. Additionally, another study demonstrated that TPC-mediated lysosomal Ca^2+^ release stimulated autophagy via Ca^2+^/calmodulin-dependent kinase kinase β (CaMKKβ) in cells over-expressing an LRRK2 mutation that causes an autosomal dominant form of PD and activates autophagy [144]. Upregulation of a mutated form of LRRK2 was associated with rising cytosolic Ca^2+^ concentrations due to increased TPC-mediated Ca^2+^ efflux from the acidic organelles (reviewed by [107]). The role of the lysossomal Ca^2+^ channels in neurodegenerative disorders is summarized in Table 1. 

Previous work has shown that lysosomal Ca^2+^ release can be mediated by the activation of transient receptor potential cation channel, mucolipin subfamily, member 1 (TRPML1), TRPML3, and transient receptor potential cation channel, melastatin subfamily, member 2 (TRPM2) (reviewed by [146]). Each of these channels has been implicated as a possible regulator of autophagy, and their dysregulation may be involved in several degenerative diseases [147]. More specifically, lysosomal Ca^2+^ release mediated by TRPML1 regulates the subcellular localization of transcription factor EB (TFEB), a member of the MiT-TFE helix–loop–helix leucine-zipper (bHLH-Zip) family of transcription factors, that, once in the nucleus, binds to the coordinated lysosomal expression and regulation (CLEAR) sequence, transcriptionally regulating the expression of TRPML1, as well as other autophagic and lysosomal genes [148,149]. TRPML1-mediated lysosomal Ca^2+^ release activates CaN, a phosphatase that binds and dephosphorylates TFEB. Furthermore, TRPML1 overexpression was shown to induce autophagy, whereas TRPML1 silencing attenuated autophagy in HeLa cells [148]. 

Under nutrient-rich conditions, serines (S122, S142, S211) of TFEB are phosphorylated by mammalian/mechanistic target of rapamycin (mTOR) [150], and S142 has also been shown to be phosphorylated by extracellular signal-regulated kinases 2 (ERK2) [151]. When S211 is phosphorylated, TFEB interacts with chaperone 14-3-3 and masks the nuclear localization signal (NLS), causing cytoplasmic retention [152]. During amino acid deprivation, exercise, or lysosomal stress, mTOR activity is inhibited, and TFEB is dephosphorylated by CaN and rapidly translocated to the nucleus, promoting the transcriptional activation of target genes. Notably, several studies have shown that TRPML1-mediated Ca^2+^ release is necessary for the activity of mTORC1 [148,153] that interacts with lysosomes and, in the presence of amino acids, is located on peripheral lysosomes [154].

Located in early and late endosomes/lysosomes, TRPML3 has also been associated with autophagy regulation. In HeLa cells, it was demonstrated that TRPML3 overexpression potentiated, and channel silencing inhibited autophagy, and expression levels of this channel also influenced endocytosis and membrane trafficking [155].

TRPM2 is primarily located in the plasma membrane but is also expressed at the surface of lysosomes, where channel-mediated Ca^2+^ release is stimulated by adenosine diphosphoribose [156], hydrogen peroxide [157,158], as well as other stimuli [159]. Notably, the NAADP concentration required for promoting lysosomal TRPM2-mediated Ca^2+^ release is much higher than the values reported for other channels stimulated by this second messenger. Currently, the physiological relevance of NAADP-mediated TRPM2 activation is still unclear [160]. In addition, TPC isoforms have been proposed to be NAADP sensitive and are capable of inducing endolysosomal Ca^2+^ release [139]. Recent findings have demonstrated that NAADP-mediated autophagy activation via TPCs occurs in rat astrocytes [161], neurons [144,162], and hepatocytes [163]. Additionally, TPC overexpression or treatment with NAADP-AM, a membrane-permeable form of NAADP, both stimulated autophagy [161,162], and cells transfected with a TPC2 mutant showed reduced light chain 3 (LC3-GFP) puncta, suggesting that TPC2 is participating in fusion events during autophagy [161]. Interestingly, in cells overexpressing an LRRK2 mutation, associated with an autosomal dominant form of PD, TPC-mediated lysosomal Ca^2+^ release was shown to be responsible for autophagy activation via CaMKKβ (reviewed by [144]). 

The physiological importance of the TRPML1-mediated Ca^2+^ release from late endosomes and lysosomes [164] is perhaps most evident in cases of mucolipidosis type IV. This neurodegenerative lysosomal storage disorder is an etiological factor in dysfunctional TRPML1 channel activity and characterized as an impairment in lysosomal pH; the accumulation of ubiquitin proteins, sequestosome-1 (SQSTM1), and undigested material; enlarged endosomal/lysosomal compartments and the presence of autophagosomes, emphasizing the consequences of defective autophagic-lysosomal function [165].

In cases of early-onset AD, presenilin 1 mutations alter lysosomal activity, impairing V-ATPase-mediated lysosomal acidification, and perturbing Ca^2+^ homeostasis [166]. In cases of familial AD mutations in the APP, presenilin 1 and 2 genes, subsequent Aβ accumulation was reported [167], and, in embryonic fibroblasts from presenilin-double knockout mice, TPC1 and TPC2 expression levels were altered, and lysosomal Ca^2+^ concentrations were reduced [168]. Therefore, it appears as though presenilin is involved in TPC regulation (Table 1). 

With regards to the familial α-synuclein PD models, it was recently demonstrated that TRPML1 agonists promoted lysosomal exocytosis, reduced α-synuclein secretion, and prevented α-synuclein accumulation in a human neuroglioma cell line. Thus, demonstrating the promising therapeutic potential of specific channel activating compounds could contribute to the treatment of PD [169].

Previous studies have shown that TRPML1 is an important component of lysosomal Ca^2+^ release, lysosomal storage, vesicle trafficking, and lysosomal acid homeostasis, and the dysregulation of these processes has been associated with several degenerative disorders [151,170,171]. For example, in an AD animal model, Zhang et al. (2017) detected downregulated TRPML1 expression and observed that recognition and memory impairments were reversed with TRPML1 overexpression [172]. Using Gly–Phe–β-naphthylamide (GPN) to induce lysosomal Ca^2+^ release, Coen et al. (2012) demonstrated that the content of lysosomal Ca^2+^ stores was significantly reduced in presenilin gene deleted cells and neurons [173]. This result is particularly relevant since the majority of early-onset familial AD cases are related to presenilin mutations [174]. 

## 3. The Role of Ca^2+^ in Autophagy

Autophagy has evolved to become a lysosomal catabolic process, allowing cells to degrade and recycle deleterious components or organelles by autophagosomes and preserve cell homeostasis. Under normal physiological conditions, autophagy activation is maintained at basal levels but can be induced in response to nutrient deprivation, hypoxia, ER stress, and DNA. Autophagy supplies the cells with the essential amino acids, nucleotides, and fatty acids, needed for the synthesis of new molecules. However, post-mitotic cells, such as neurons, have a low proliferative rate and depend heavily on autophagy for cellular maintenance, making them extremely vulnerable to changes in this pathway damage (reviewed by [175]).

Three major pathways of autophagy have been identified and include macroautophagy, microautophagy, and chaperone-mediated autophagy (CMA) [176,177]. Macroautophagy, hereafter-called autophagy, is a process initially characterized by the formation of autophagosomes [178]. The autophagosomes fuse with lysosomes to form autophagolysosomes—in a Ca^2+^-sensitive manner—which is in contrast to microautophagy or CMA, where cargos are sequestered and internalized with or without the aid of lysosomal membrane receptors (reviewed by [179]).

Autophagy is directly related to anabolic and catabolic processes, participating in a variety of regulatory pathways involved in cell growth and proliferation. In fact, the phosphatidylinositol 3-kinase/protein kinase B (PI3K/AKT) and MAPK pathways are typically activated by the mechanistic target of rapamycin complex 1 (mTORC1) (reviewed by [180]), the master regulator of the canonical autophagic response. In contrast, nutrient deprivation activates the adenosine monophosphate-activated protein kinase (AMPK) pathway, which is dependent on cytosolic Ca^2+^ levels and Ca^2+^ dependent kinases, such as Ca^2+^/calmodulin-dependent kinase 2 (CaMKK2), and blocks mTORC1, ultimately inducing autophagy [181].

The ATG proteins have also been shown to participate in the activation of autophagy, modulating the machinery responsible for autophagosome formation. Previous studies have shown that AMPK-mediated Ca^2+^ release leads to ATG1 protein Unc-51 like autophagy activating kinase (ULK1) activation and autophagy induction [181,182]. Together with the Vps34 complex and under the control of the Atg12-Atg5-Atg16L complex, the activated ULK1 complex induces phagophore isolation and autophagosome membrane elongation. Concomitantly, LC3, a protein that participates in the cargo recognition, autophagosome membrane closure, and maturation, is lipidated and conjugated to phosphatidylethanolamine, forming the LC3-II (reviewed by [183]). It has been suggested that autophagy selectivity is mediated by the ubiquitination of targets that need to be degraded, which are subsequently recognized by autophagic adapters, such as sequestosome-1 (SQSTM1/p62), and bound to LC3-II [184].

**Figure 2:**
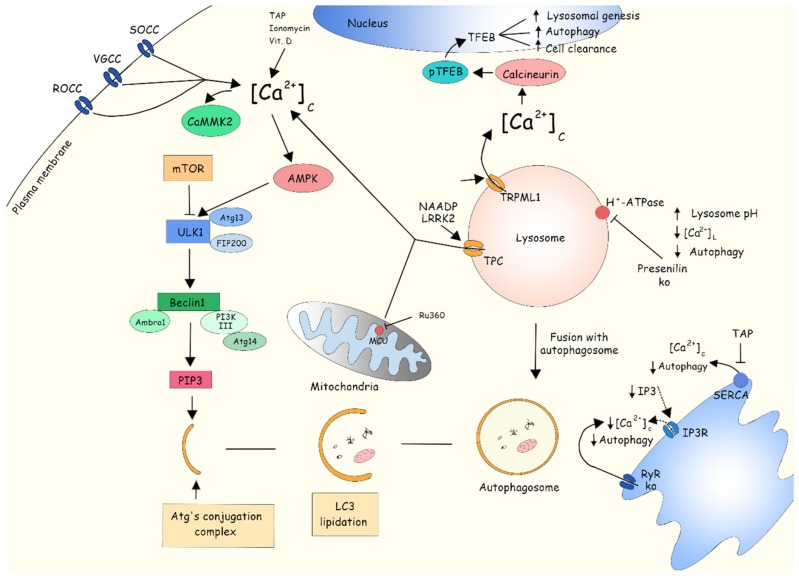
Ca^2+^ is mostly involved in mTOR-independent autophagy induction. The canonical pathway of regulation and induction of autophagy is controlled by the mammalian/mechanistic target of rapamycin (mTOR). A variety of factors can inhibit mTOR and lead to autophagy activation. Inactivated mTOR activates and phosphorylates Unc-51 like autophagy activating kinase (ULK1) complex, forming a multiprotein complex with Beclin1, VPS34, AMBRA1, VPS34, and PI3K-III, known as the PI3K class III complex, and the formation of PIP_3_ (phosphatidylinositol 3-phosphate). PIP_3_ induces the initiation of the phagophore membrane, while the elongation is controlled by the Atg12-Atg5-Atg16L complex. At the same time, LC3 is lipidated and conjugated to phosphatidylethanolamine, forming the LC3-II and participating in the cargo recognition and closure of the autophagosome membrane. Cargo is degraded by the fusion of autophagosomes and lysosomes and is performed by lysosomal hydrolases. Ca^2+^ physiology and signaling have an important role in the induction of autophagy. Increased cytosolic Ca^2+^ levels, mediated by SOCC or release of intracellular stores (induced by thapsigargin, vitamin D ionomycin, or Ru360), can activate Ca^2+^calmodulin dependent kinase 2 (CaMKK2), which will activate protein kinase B (AKT); or alternatively, Ca^2+^ can directly activate AKT, initiating the signaling processes necessary for autophagy induction. The Ca^2+^ released from the lysosome can also activate AKT and CaMKK2, by the stimulating two-pore channels (TPC) and transient receptor potential cation channel, mucolipin subfamily, member 1 (TRPML1). In PD, the mutated protein leucine-rich repeat kinase 2 (LRRK2) can upregulate TPC activity, causing increased Ca^2+^ transport through the channel, and augment autophagy. The stored lysosomal Ca^2+^ released via TRPML1 activates calcineurin, which leads to transcription factor EB (TFEB) dephosphorylation. Dephosphorylated TFEB translocates to the nucleus, regulating lysosomal genesis, activation of autophagy, and increasing cell clearance. On the other hand, reduced Ca^2+^ channel activity can attenuate the autophagic response. Knocking-out presenilin leads to reduced lysosomal H^+^-ATPase pump and SERCA activities, as well as reduced autophagy. SERCA inhibition with thapsigargin shows similar results. Attenuated IP_3_R activity inhibits Ca^2+^ release from the ER, consequently reducing the CICR via RyR activation and decreases the autophagic response.

The first study investigating the role of Ca^2+^ in autophagy was performed by Gordon et al. (1993) and demonstrated that extracellular and intracellular Ca^2+^ chelators (BAPTA-AM) reduced the levels of autophagy in isolated rat hepatocytes [185]. However, Høyer-Hansen et al. (2007) later showed that cytosolic Ca^2+^ mobilizing agents (i.e., vitamin D3, thapsigargin, or ionomycin) induced autophagosome accumulation, which was accompanied by mTORC1 inhibition and CAMKK2 and AMPK activation [186]. Indeed, several reports have shown that Ca^2+^ can exert both inhibitory and stimulatory effects on autophagy [187,188], and the intensity of the Ca^2+^-mediated autophagy response depends on both the amplitude of the response and the specific channels involved (Figure 2).

As described above, Ca^2+^-permeable ion channels are present in Ca^2+^-storing organelles, such as the ER, mitochondria, and lysosome, as well as in the plasma membrane. In the ER, IP_3_R activation was shown to result in both stimulatory and inhibitory processes. Initially, IP_3_Rs were postulated to function as autophagy inhibitors since low IP_3_ levels increased autophagy [189], an observation that was further verified using IP_3_ antagonists [190] receptor silencing [191]. 

Recent studies have shown that ER-mitochondrial contact sites are directly involved in starvation-induced autophagy. For example, it has been shown that ATG14L and ATG5 proteins are recruited to these contact sites at the beginning of autophagosome formation [192]. Due to the fact that IP_3_Rs are also present in these contact sites, it is reasonable to hypothesize that these channels are also involved in this process. Several studies have investigated the necessity of IP_3_R in autophagy, with one study reporting that starvation leads to IP_3_R sensitization through an enhanced association with Beclin-1 and that the Beclin-1 knockdown prevents IP_3_R-mediated Ca^2+^ release from starved cells. Additionally, the IP_3_R inhibitor xestospongin B, as well as BAPTA-AM, abolished autophagy induction [187]. It has been suggested that IP_3_R could also play a role in autophagic flux since IP_3_R antagonists rapidly and completely blocked lysosomal Ca^2+^ uptake through ER-lysosome contact sites, thereby resulting in lysosomal dysfunction [193]. Taken together, these data indicate that IP_3_R could regulate autophagy at different steps. 

With regard to RyR-regulated autophagy, several studies have shown that RyR-mediated signaling culminates in autophagy inhibition. In murine cardiomyocytes, Yuan et al. (2014) demonstrated that deleting Fkbp1b resulted in RyR leakage and mTOR induction [194]. Furthermore, cardiac cells from ryanodine receptor 2 (RYR2) knock-out mice were found to present reduced mitochondrial Ca^2+^ concentrations and signs of autophagy activation [195]. In C2C12 myoblasts and primary hippocampal neurons, the RyR inhibitor dantrolene increased lysosomal turnover and augmented autophagic flux [196]. 

The role of SERCA in autophagy induction is poorly understood. Studies showed that thapsigargin treatment, which increases cytosolic Ca^2+^ levels with a concomitant loss of ER Ca^2+^ stores, blocked autophagic flux and inhibited autophagosome and lysosome fusion [197]. Indeed, it is, therefore, plausible that the thapsigargin-related stimulatory and inhibitory effects on autophagy are related to Ca^2+^ levels. This compound has been shown to inhibit nutrient starvation-induced autophagy [198]. Additionally, Zhao et al. (2017) showed that SERCA activity could be modulated by vacuole membrane protein 1 (VMP1) protein, promoting the dissociation of ER membrane contacts, which are essential for autophagosome formation [199].

With regards to MCU, data suggested that it negatively regulated basal autophagy rates since the pharmacological agent Ru360 and genetic inhibition of the uniporter abrogated mitochondrial Ca^2+^ uptake increased AMP:ATP ratios, activated AMPK, and induced mTORC1 independent autophagy [200,201,202].

It is well known that VGCC has a regulatory role in autophagy; however, the specific function of these channels in autophagy is still unclear and likely depends on the type of channel activated or inhibited. For example, L-type Ca^2+^ channel antagonists (verapamil, loperamide) increase autophagy independent of mTORC1, while L-type Ca^2+^ channel agonist BAYK8644 activates calpains and increases cAMP levels, which, in turn, positively regulates IP_3_ levels and results in increased cytosolic Ca^2+^ levels. As a result of continuous calpain activation, a cyclic pathway of autophagy inhibition is created [203].

Several intracellular processes, including apoptosis, cellular proliferation, and gene expression, involve SOCC, and the available data in the literature suggests that these channels can differentially influence autophagy depending on cell type and autophagy inducer (reviewed by [204]). Moreover, inhibition of SOCC-mediated Ca^2+^ entry by SKF-96365 was shown to stimulate autophagy through AKT/mTORC1 pathway inhibition [205,206].

On the other hand, studies have also demonstrated that Ca^2+^ entry via SOCC positively regulates autophagy. In endothelial progenitor cells, increased Ca^2+^ transport through SOCC activated autophagy via the CAMKK2/mTOR pathway, and both Ca^2+^ chelators (EGTA or BAPTA-AM) and STIM1-silencing inhibited autophagy, thus confirming the involvement of SOCC in this process [207]. Under hypoxia, mimicked by dimethyloxalylglycine (DMOG) and desferrioxamine (DFO) agents, SKF-96365 effectively inhibited autophagy, an effect attributed to the inhibition of TRPC1 [208], which is also a molecular component of SOCCs [209]. Therefore, taking into consideration the discrepancies in the literature from different groups, further studies are required for elucidating the role of SOC channels in autophagy.

The study results presented up to this point clearly demonstrates channel diversity and emphasizes the importance Ca^2+^ signaling has on autophagy regulation, with evidence suggesting that cellular Ca^2+^ signals are involved at different stages of the autophagy process, and playing roles in the initiation of phagophore formation and elongation and autophagosome/endosome fusion with the lysosome. Ca^2+^ signaling at ER-mitochondria contact sites would likely influence autophagy initiation, whereas Ca^2+^ channel activity at ER-lysosome contact sites would modulate lysosomal function, autophagosome fusion, and cargo degradation. For instance, with respect to AD, the deletion of presenilin-1 leads to a loss of lysosomal vATPase activity, which raises lysosomal pH, perturbs TRPML-mediated lysosomes, and blocks autophagy [210].

Given the diversity of cellular Ca^2+^ signaling and Ca^2+^ sources, the role this ion plays in autophagy is extremely complex and relies on multiple factors, such as cell type, status, including nutritional, along with ion channel type, localization, expression levels, isoforms, and regulatory mechanisms. Indeed, ion channel dysfunctions and altered autophagy regulation have been implicated in various diseases, suggesting that Ca^2+^ channel expression and/or activity could be exploited as potential therapeutic targets for the modulation of autophagic processes.

In PD, the presence of Lewy bodies formed by aggregated α-synuclein is probably the result of protein aggregates that are resistant to autophagic degradation [211]. Accordingly, the presence of these aggregates may also impair the autophagy pathways [212]. Mutations in both α-synuclein and LRRK2 genes are associated with late-onset PD, while mutations in PINK1 and Parkin, two genes classically involved in mitophagy, are related to early-onset PD [213,214]. LRRK2 is present in the lysosomes and controls Ca^2+^ release involving activated TPC channels. Following the release of Ca^2+^ from the lysosome, Ca^2+^ is also released from the ER, resulting in Ca^2+^/CaMKK/AMPK pathway activation and autophagy induction. The increase in LRRK2 activity and Ca^2+^ release leads to a pathological increase in autophagy induction and defective lysosomal acidification [144]. The overexpression of an LRRK2 mutant in neuronal cells decreased neurite process length, along with a concomitant accumulation of autophagic structures [215,216]. Besides impairing mitophagy, a PINK1 mutation makes neurons more vulnerable to Ca^2+^-induced cell death, representing yet another potential cause of PD pathogenesis [217].

## 4. Conclusions and Perspectives

Based on the results from a variety of experimental models, it is clear that specific AD, PD, and HD-related proteins can disrupt Ca^2+^ homeostasis; however, the exact role that Ca^2+^ plays in apoptosis and autophagy regulation, especially in neurodegenerative diseases, remains unknown. In the CNS, Ca^2+^ controls cell fate and metabolism, accounting for most of the energy expenditure of the body. Altered Ca^2+^ homeostasis has been linked to numerous pathological processes and can lead to the activation of death pathways. Therefore, Ca^2+^ levels must be well regulated under these conditions since neurons are particularly susceptible to damage. The ER, mitochondria, and lysosomes mainly control Ca^2+^ buffering, and the interplay between these organelles, along with Ca^2+^ transport mechanisms involved in shuttling this ion across membranes, are essential to cellular homeostasis, as well as the modulation of autophagy. This catabolic process emerges as a neuroprotective strategy since the basis of neurodegenerative diseases lies in the accumulation of unfolded proteins that consequently impair cell function, alter metabolism, and upregulate the unfolded protein response, to the demise of the cytoskeleton. In addition, it has been reported that during aging and in age-related neurodegenerative pathologies, there is an impairment of autophagy, accompanied by elevated oxidative stress and unbalanced energy. Understanding the role of Ca2+ regulation buffering in apoptosis and autophagy could reveal novel therapeutic targets for combating neurodegenerative diseases.

## Figures and Tables

**Table 1:** Lysosomal Ca^2+^ channels associated in neurodegenerative disorders.

Receptor	Ca^2+^ Alterations	Disease Model	Reference
**TRPML1 ^1^**	Defective autophagic-lysosomal function	Mucolipidosis type IV	[165]
**TPC2 ^2^**	Enhanced TPC2 Ca^2+^ levels; autophagy and endo-lysosomal morphology impairment	PD ^3^	[143] [144] [145]
**TPC1/2**	Altered TPC1 and TPC2 levels and reduced lysosomal Ca^2+^	AD ^4^	[168]

^1^ TRPML1 (transient receptor potential cation channel, mucolipin subfamily, member 1); ^2^ TPC (Two-pore channel); ^3^ PD (Parkinson’s disease); ^4^ AD (Alzheimer’s disease).
